# Promoter Specific Methylation of *SSTR4* is Associated With Alcohol Dependence in Han Chinese Males

**DOI:** 10.3389/fgene.2022.915513

**Published:** 2022-06-09

**Authors:** Rongrong Zhao, Huihui Shi, Jiajun Yin, Zhen Sun, Yahui Xu

**Affiliations:** ^1^ The First Affiliated Hospital and College of Clinical Medicine of Henan University of Science and Technology, Luoyang, China; ^2^ Brain Science Basic Laboratory, The Affiliated Wuxi Mental Health Center with Nanjing Medical University, Wuxi, China; ^3^ The Second Affiliated Hospital of Xinxiang Medical University, Xinxiang, China

**Keywords:** alcohol dependence, gene expression, hypomethylation, SSTR4, Han Chinese

## Abstract

Alcohol dependence (AD), a disease can be affected by environmental factors with epigenetic modification like DNA methylation changes, is one of the most serious and complex public health problems in China and worldwide. Previous findings from our laboratory using the Illumina Infinium Human Methylation450 BeadChip suggested that methylation at the promoter of *SSTR4* was one of the major form of DNA modification in alcohol-dependent populations. To investigate whether DNA methylation levels of the *SSTR4* promoter influence alcohol-dependent behaviors, genomic DNA was extracted from the peripheral blood sample of 63 subjects with AD and 65 healthy controls, and pyrosequencing was used to verify the results of BeadChip array. Linear regression was used to analyze the correlation between the methylation levels of *SSTR4* promoter and the scores of alcohol dependence scales. Gene expression of *SSTR4* in brain tissue was obtained from the Genotype-Tissue Expression (GTEx) project and Human Brain Transcriptome database (HBT). We found the methylation levels of *SSTR4* in AD group were significantly lower than healthy controls (two-tailed *t*-test, *t =* 14.723, *p* < 0.001). In addition, only weak to moderate correlations between the methylation levels of the *SSTR4* promoter region and scale scores of Alcohol Use Disorders Identification Test (AUDIT), Life Events Scale (LES) and Wheatley Stress Profile (WSS) based on linear regression analyses (AUDIT: *R* ^2^ *=* 0.35, *p* < 0.001; LES: *R* ^2^ = 0.27, *p* < 0.001; WSS: *R* ^2^ = 0.49, *p* < 0.001). The hypomethylated status of *SSTR4* may involve in the development of AD and increase the risk of AD persistence in Han Chinese males.

## Introduction

Alcohol dependence (AD) is a common chronic disorder which imposes a substantial burden on global health. According to World Health Organization (WHO) reports, there were approximately 3.3 million alcohol-related deaths worldwide in 2014, including 320,000 young individuals aged 15 to 29 (Organización Mundial de la Salud, 2014). It is estimated that more than 1.8 million persons were dependent on alcohol, and 1.6 million persons had a lifetime history of alcohol abuse in Germany (Batra et al., 2016). Family, twin and adoption studies have indicated genetic basis for AD susceptibility (Reilly et al., 2017), with the variation in heritability from a range of 40%–70% (Enoch and Goldman, 2001; Agrawal and Lynskey, 2008; Lynskey et al., 2010; Zhang et al., 2012). In addition, environmental factors may play important roles in AD development through epigenetic regulation of gene expression without DNA sequence alterations (McCutcheon et al., 2012).

Epigenetic regulatory mechanisms could induce stable changes in gene expression with a range of phenotypic outcomes via DNA methylation, histone acetylation, chromatin remodeling, and noncoding RNA regulation (Kouzarides, 2007; Krishnan et al., 2014). Cytosine methylation at CpG dinucleotides-rich regions (CpG islands) is the common epigenetic modification found in DNA where the methylation plays a pivotal role in mediating gene transcription regulation by affecting transcription factor binding. Numerous studies have indicated although most genomic CpGs were stably methylated, CpG islands near or within the promoter regions maintained commonly low methylation levels to allow the transcriptional activation of related gene dynamically, and its dysregulated methylation contributed to disease progression in cases of environmental challenges (Egger et al., 2004; Moore et al., 2013). It was thought that disturbances of epigenetics also participate in pathophysiological processes of AD (Basavarajappa and Subbanna, 2016). Other studies have also found hypomethylation of several genes such as *GDAP1* correlated with increased alcohol consumption (Brückmann et al., 2016), and elevated N-methyl-D-aspartate 2b receptor subunit gene (Biermann et al., 2009) and proopiomelanocortin gene (Muschler et al., 2010) promoters methylation was detectable in DNA from peripheral blood of patients with AD. These alterations in DNA methylation might impact the transcriptional profile and the susceptibility to AD (Wilson et al., 2019). For example, in animal models of AD, up-regulation of *Gdnf* expression due to altered methylation of core promoter or negative regulatory element has been observed in Nucleus Accumbens, which are key brain regions associated with reward and addictive behaviors (Maier et al., 2020). Moreover, specific genetic variants at methylation quantitative trait loci might also influence AD susceptibility via altering DNA methylation status (Zhang et al., 2014). However, evidence from laboratory-based data may not be conclusive, and epidemiological studies are required to better understand the biological mechanisms of alcohol addiction, which could aid in the clinical treatment or prevention of AD.

Somatostatin receptor 4 (SSTR4) is a brain-specific G-protein-coupled receptor as known substrate of somatotropin-release inhibitory factor implicated in the pathophysiological processes of anxiety and depression-like behavior (Günther et al., 2018). Previous studies have shown *SSTR4* is expressed in areas involved in learning and memory processes, and the activation of hippocampal *SSTR4* leads to a switch from hippocampus-based memory to dorsal striatum-based behavioral responses (Gastambide et al., 2009). In addition, experimental data suggest *SSTR4* might represent important therapeutic targets for the treatment of Alzheimer’s disease and seizures, yet the direct evidence for the role of *SSTR4* in alcoholism is still lacking.

Our previous genome-wide study based on methylation detection utilizing the Illumina Infinium Human Methylation450 (Illumina Inc., San Diego, California) on DNA extracted from peripheral blood (PB) of 10 AD subjects and 10 paired siblings without AD revealed 1,581 differentially methylated CpG positions (including 865 hypomethylation islands and 716 hypermethylation islands), which were associated with 827 well-annotated reference genes (Zhao et al., 2013). Our data suggested novel potential epigenetic targets relevant to AD. DNA pyrosequencing technology has also been to examine the 2 top-ranked hypo or hypermethylation AD-related genes from Illumina microarrays determined by DAVID. Linear regression analysis showed good correlation between DNA microarrays and pyrosequencing results. In alcohol-dependent subjects, the most prominent hypomethylated CG dinucleotide sites were located in the promoter of *SSTR4*. The objective of current research was to validate the demethylated status of *SSTR4* in Han Chinese alcohol-dependent males.

## Materials and Methods

### Subjects

The current research utilized clinical and methylation microarrays (Illumina Infinium Human Methylation450) data extracted from our previous analyses and newly recruited subjects. This study was approved by the Ethics Committee of the Second Affiliated Hospital of Xinxiang Medical University (2015 Ethics number 27), and written or oral informed consent was obtained from each participant. Blood samples of validation cohort included 128 male participants (63 AD and 65 healthy controls) recruited from community or medical clinic settings of northern Henan Province. A consistent diagnosis of AD was made by at least two psychiatrists according to the criteria of the Diagnostic and Statistical Manual of Mental Disorders, 4th edition (DSM-Ⅳ) (American Psychiatric Association, 1994). The Alcohol Use Disorders Identification Test (AUDIT, score range 0–40) was utilized to measure quantity-frequency of alcohol consumption, and the score of AUDIT greater than or equal to 8 suggested and problematic drinking and AD tendency (Babor et al., 2001). The Life Events Scale (LES) (Trivedi et al., 2010) and Wheatley Stress Profile (WSS) (Wheatley, 1990) were used to assess negative life events and possible stress factors associated with AD. Controls were screened to exclude those with alcohol or drug abuse or dependence. We also ruled out subjects with other substance misuse, comorbidity in major psychiatric disorders, serious medical complications, severe neurological or somatic illnesses.

### DNA Extraction and Amplification

The QIAmp DNA Blood Mini Kit (Qiagen, Hilden, Germany) was utilized to extract and purify genomic DNA from PB. Forty microliters of DNA solution were treated with the CT conversion reagent included in the EpiTect Plus LyseAll Bisulfite Kit (Qiagen, Hilden, Germany) according to the manufacturer’s protocol. The concentration of bisulfite-treated DNA was determined using a Thermo Nanodrop 2000 spectrophotometer, and the DNA volume was determined to be at least 3 μl. Because the nucleotide composition of DNA is changed and the DNA fragments are smaller after bisulfite conversion, the results of subsequent experiments are not ideal. Therefore, the whole genome was amplified after bisulfite transformation, and the sequence after transformation was maintained. One hundred nanograms of bisulfite-treated DNA were amplified using an EpiTect Whole Bisulfitome Kit (Qiagen, Hilden, Germany) under the following conditions: 8 h at 28°C and 5 min at 95°C.

### Pyrosequencing

Primers were designed using Pyrosequencing Assay Design Software (Biotage AB, Uppsala, Sweden): *SSTR4*1f (PCR forward, biotin-labeled), 5′-TTT​TTG​GAG​TTT​AGT​AGA​AGA​AGG​GTA​AT-3’; *SSTR4*1r (PCR reverse), 5′-CAC​CCT​ATA​ACC​TAA​TTC​AAT​CAT​TAT​C-3’; S*STR4*1s (PCR sequencing), 5′-ATC​CCT​AAC​CAC​TAA​AAT​A-3’. PCR was performed using 10 ng of bisulfite-treated DNA using a PyroMark PCR Kit (Qiagen, Hilden, Germany) under the following conditions: the Initial PCR activation step was 15 min at 95°C, followed by 50 cycles of 30 s at 94°C (denaturation), 30 s at 56°C (annealing), 30 s at 72°C (extension), and a final extension of 10 min at 72°C.

Pyrosequencing was performed using PyroMark Gold Q96 Reagents (Qiagen, Hilden, Germany). We applied the Biotage PyroMark MD System (Biotage) to conduct pyrosequencing reactions via sequential nucleotide additions in the predetermined orders based on the instructions of manufacturer. RAW sequencing data were quantitatively analyzed by using Pyro Q-CpG 1.0.9 software (Biotage). The methylation levels of CpG regions were assessed by the percentage of methylated cytosines (M) over the total methylated and unmethylated cytosines (M + U) in the genome.

### Statistical Methods

**FIGURE 1:**
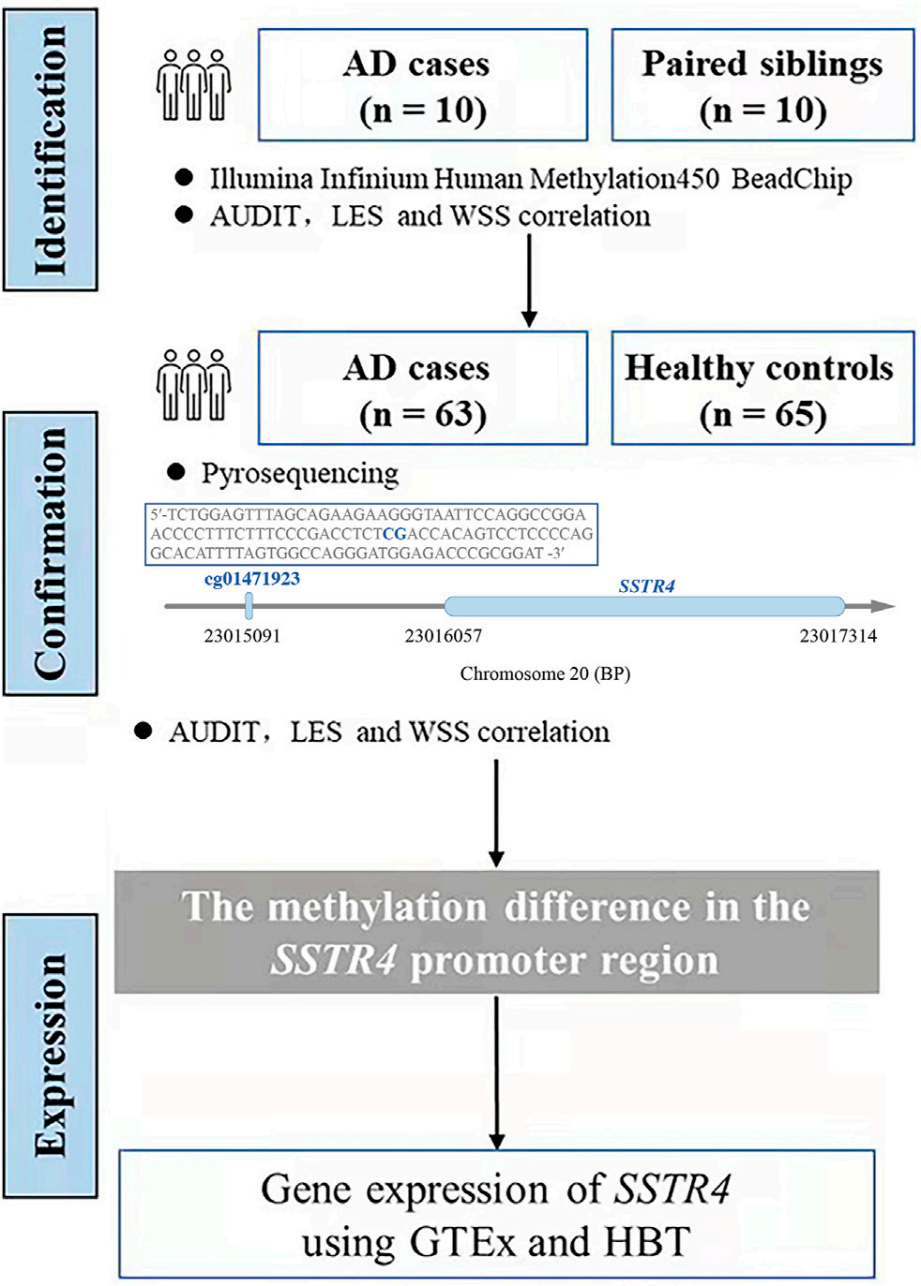
The flowchart of research. We used the sequence in the box by pyrosequencing to examine the methylation level of cg01471923 (chr20:23015091), a CpG site located at the promoter region of gene SSTR4.

The DNA methylation microarray from Illumina were utilized in reference to our previous findings. DNA methylation levels between 10 AD subjects and 10 paired siblings were compared using the two-tailed paired Student’s t-test based on the unequal variance assumption. The methylation value of the *SSTR4* promoter region in validation cohort (63 subjects with AD and 65 healthy controls) was analyzed using a two-tailed unpaired *t*-test with unequal variance. Linear regression analysis was used to examine the associations between the methylation levels of the *SSTR4* promoter region and scale scores of AUDIT, LES and WSS. Gene expression of *SSTR4* was confirmed by the Genotype-Tissue Expression database (GTEx, www.gtexportal.org) and Human Brain Transcriptome database (HBT, www.hbatlas.org) (Kang et al., 2011; Ramasamy et al., 2014; GTEx Consortium, 2015). Two-tailed *p* value less than 0.05 were considered statistically significant. An overview of subject recruitment and promoter methylation levels analysis was presented as a flow chart in Figure 1.

## Results

### The Correlation Between the Methylation Levels of the *SSTR4* Promoter Region in Blood and the AUDIT, LES, WSS Scores

**FIGURE 2:**
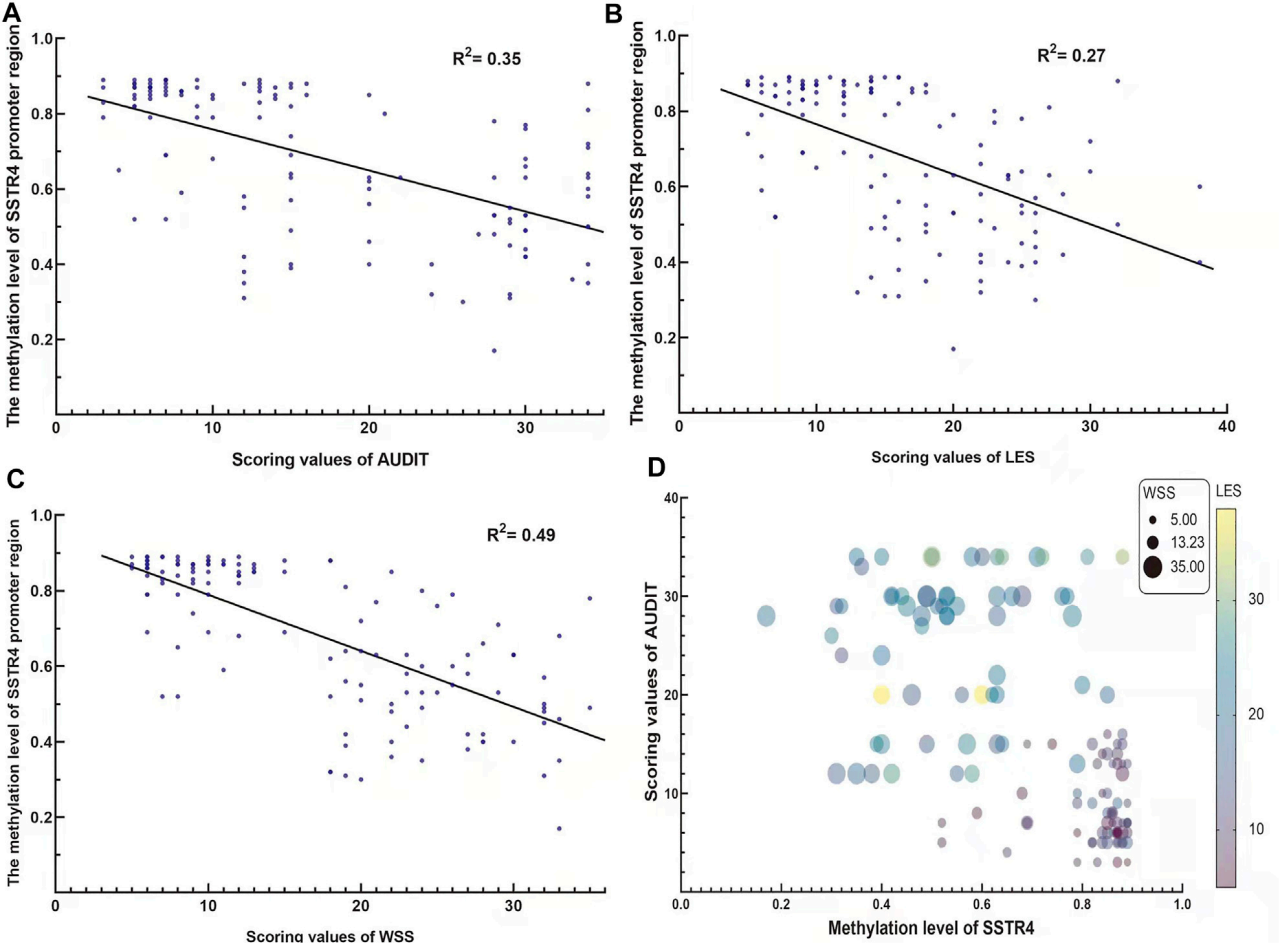
The correlation between the methylation value of the *SSTR4* promoter region and AUDIT, LES and WSS scores. **(A)** The methylation value of the *SSTR4* promoter region and the AUDIT score were negatively correlated. **(B)** The methylation value of the *SSTR4* promoter region and the LES score were negatively correlated. **(C)** The methylation value of the *SSTR4* promoter region and the WSS score were negatively correlated. **(D)** Bubble plot for the visualization of association of SSTR4, AUDIT4, LES and WSS.

Considering potential gender effect on genome-wide DNA methylation, only male subjects were recruited in this study. Overall, the mean age of subjects were similar between AD group (mean age 39.1 ± 7.3) and healthy control (mean age 39.6 ± 8.1), with no significant difference (*p* = 0.722). The scale scores of AUDIT, LES and WSS were higher for AD subjects than for the control group (25.4 ± 7.4 vs. 8.4 ± 3.7, 22.2 ± 5.6 vs. 10.2 ± 3.5, 25.2 ± 5.1 vs. 9.2 ± 2.9, respectively, *p*-value < 0.001). Analysis results were summarized in Table 1. Linear regression analysis revealed the methylation levels of *SSTR4* were only weak to moderate correlations with the scores of AUDIT, LES and WSS as shown in Figure 2 (AUDIT: *R* ^2^ *=* 0.35, *p* < 0.001; LES: *R* ^2^ = 0.27, *p* < 0.001; WSS: *R* ^2^ = 0.49, *p* < 0.001).

**TABLE 1:** Basic characteristic of the study population.

Feature characteristic	AD cases (*n* = 63)	Healthy controls (*n* = 65)	*t*	*p*
Age (mean ± S.D)	39.1 ± 7.3	39.6 ± 8.1	0.357	0.722
AUDIT (mean ± S.D)	25.4 ± 7.4	8.4 ± 3.7	16.301	<0.001
LES (mean ± S.D)	22.2 ± 5.6	10.2 ± 3.5	14.441	<0.001
WSS (mean ± S.D)	25.2 ± 5.1	9.2 ± 2.9	21.779	<0.001

S.D, standard deviation.

### The Methylation Difference in the *SSTR4* Promoter Region in 10 Paired Siblings Using Microarray Compared to Case-Controls Eith Pyrosequencing

**FIGURE 3:**
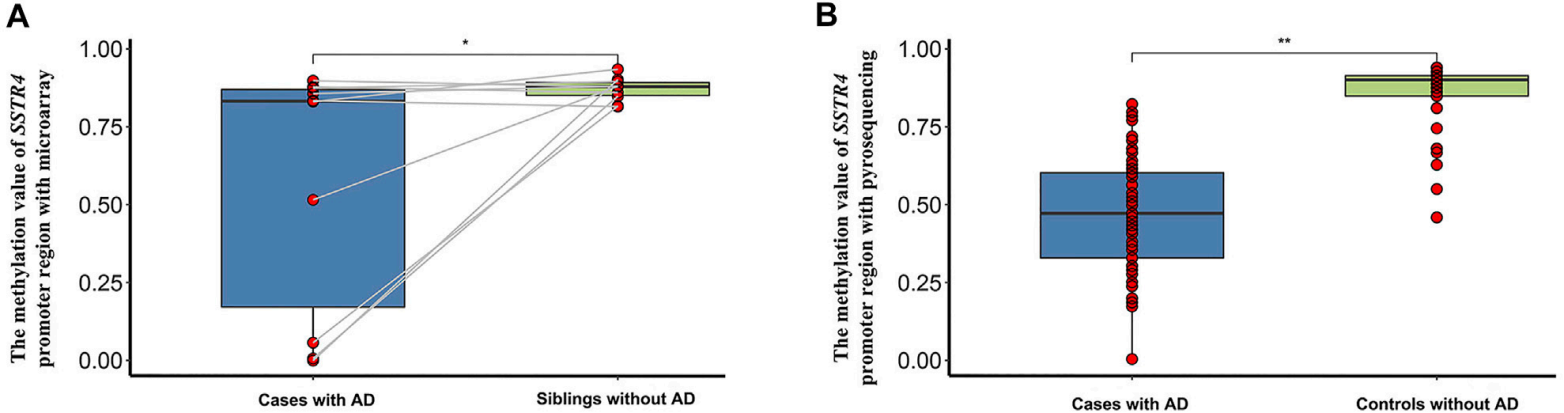
The methylation difference of the SSTR4 promoter region in 10 paired siblings with microarray and case-controls with pyrosequencing. **(A)** Box plot of the methylation value of the SSTR4 promoter region with microarray and 10 paired siblings. **(B)** Box plot of the methylation value of the SSTR4 promoter region with pyrosequencing and case-controls. ^∗^ *p* < 0.05,^∗∗^ *p* < 0.001.

The results of previous DNA methylation microarrays showed that the level of methylation of the *SSTR4* promoter region between cases and paired siblings was statistically significant (*t* = 2.348, *p* = 0.043, Figure 3A). Likewise, the level of methylation of the *SSTR4* promoter region confirmed by pyrosequencing in cases and controls was statistically significant (*t* = 14.723, *p* < 0.001), and hypomethylation of the *SSTR4* promoter region was observed in AD cases (Figure 3B).

### Gene Expression of *SSTR4* Using GTEx and HBT

**FIGURE 4:**
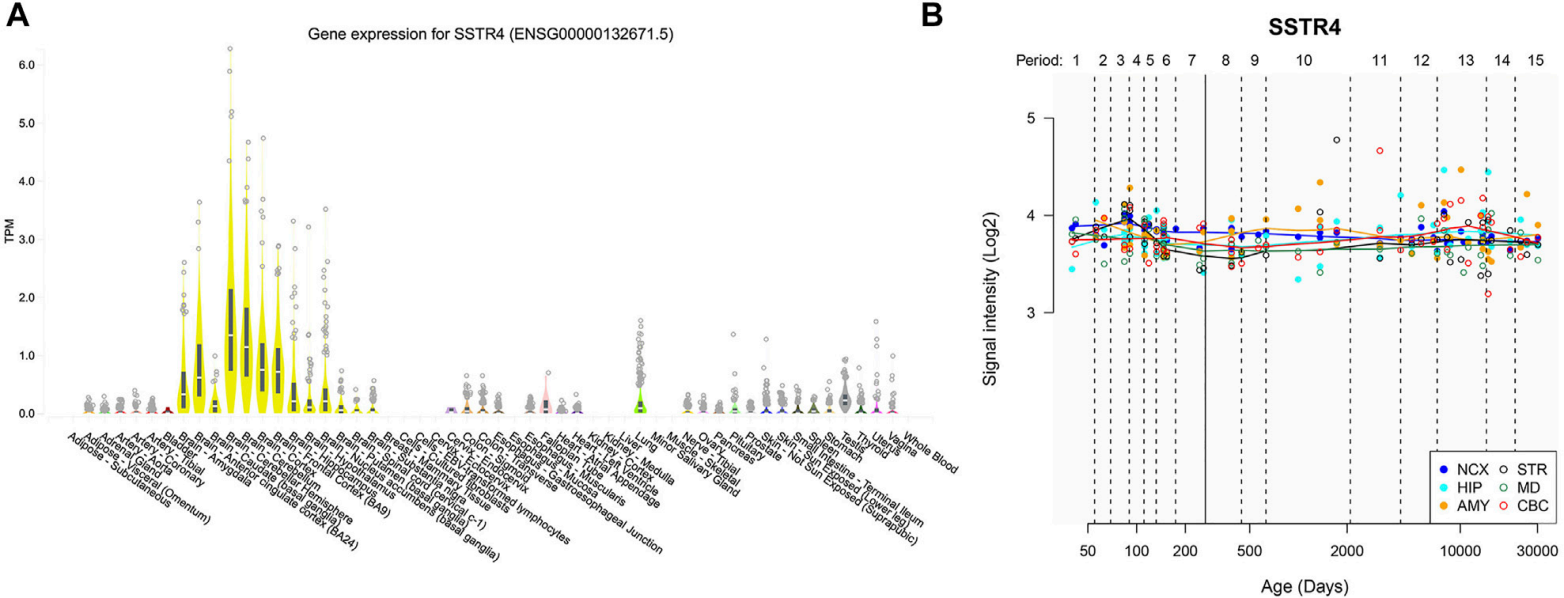
Gene expression of *SSTR4* by GTEx and HBT. **(A)** Spatial expression pattern of the *SSTR4* gene in human brain regions from GTEx. TPM = transcripts per kilobase million. Expression threshold: >0.1 TPM and ≥6 reads in 20% or more of samples. Box plots are shown as median and 25th and 75th percentiles; points are displayed as outliers if they are above or below 1.5 times the interquartile ranges. Data Source: GTEx Analysis Release V8 (dbGaP Accession phs000424. v8. p2). **(B)** Dynamic expression pattern of the *SSTR4* gene in 6 human brain regions across lifespan from HBT. NCX, neocortex; CBC, cerebellar cortex; MD, mediodorsal nucleus of the thalamus; STR, striatum; AMY, amygdal; HIP, hippocampus. Period 1, Embryonic development; Period 2, Early fetal development; Period 3, Early fetal development; Period 4, Early mid-fetal development; Period 5, Early mid-fetal development; Period 6, Late mid-fetal development; Period 7, Late fetal development; Period 8, Neonatal and early infancy; Period 9, Late infancy; Period 10, Early childhood; Period 11, Middle and late childhood; Period 12, Adolescence; Period 13, Young adulthood; Period 14, Middle adulthood; Period 15, Late adulthood.

Expression of *SSTR4* in various tissues revealed relatively strong expression in brain tissue. Although *SSTR4* is highly expressed in the cerebellar hemisphere and cerebellum, it is moderately expressed in the nucleus accumbens (NAC), prefrontal cortex (PFC), amygdala (AMY) and hippocampus (HPC) (Figure 4A). These regions are related to the reward pathway of addiction in the brain. Temporal expression analyses showed that the expression level of *SSTR4* was relatively stable across lifespan (Figure 4B).

## Discussion

These findings revealed that compared to controls, AD patients experienced more negative life events (LEs) and higher stress levels, which indicated that environmental factors play a role in the formation and maintenance of AD. This result is also consistent with our previous clinical research of 10 AD cases and 10 paired siblings without AD as controls (Zhao et al., 2013). The study of Linda Azucena Rodríguez Puente et al. (2019) showed that stressful events occur that have the potential to trigger the consumption of substances, such as alcohol. Stressful events are greater in those who consume alcohol than in those who do not consume alcohol. Likewise, the study of Marketa Krenek showed that although alcohol use severity did not predict changes in recent LEs, the emergence of LEs is associated with subsequent increases in drinking severity. This article also provided partial support for the hypothesis that distal LEs influence changes in both LEs and heavy alcohol use over time (Krenek et al., 2017). Ethan H indicated that although LEs may not necessarily contribute to the maintenance of long-term alcohol abuse among heavy drinkers with high addiction severity, daily stressful events predicted increases in daily drinking the whole time for all heavy drinking, and stress may influence the emergence of early drinking behaviors (Mereish et al., 2018). These studies’ findings were consistent with our research.

In addition, the follow-up results of our study revealed that the lower the methylation value of *SSTR4* was, the higher the AUDIT, LES and WSS values were. According to this result, stressful events (higher values of LES and WSS) may contribute to alcohol use disorder and AD (higher value of AUDIT), and then influence the methylation of *SSTR4* (hypomethylation). In contrast, hypomethylation of *SSTR4* may induce addictive behavior. It can be inferred that stressful events that lead to the hypomethylation of *SSTR4* mediate alcohol abuse. A study by Scheich et al. revealed that activation of *SSTR4* in the central nervous system plays a role in modulation of behavioral responses to acute stress and neuroendocrine changes induced by mild chronic stress in mice, suggesting involvement of *SSTR4* in anxiety and depression-like behavior (Scheich et al., 2016; Scheich et al., 2017), consistent with our research. Through these studies, we can better understand how LEs and higher stress act as high risk factors for AD. This result offers treatment options for reducing the negative effect on LEs and higher stress to reduce the germination and maintenance of AD.

AD and drugs of abuse have a moderate to high heritability component (Goldman et al., 2005). In addition to the variation of basic sequences, epigenetic modification of gene sequences may also be associated with substance dependence (Zhang et al., 2012). The present study suggested that there was significantly lower DNA methylation of the *SSTR4* promoter region in AD cases than in healthy controls. Sample sizes of AD cases and controls were increased to perform theoretical verification, which was used to confirm the results based on the research of 10 AD cases and 10 paired siblings without AD as controls.

Somatostatin (SST), also known as somatotropin-release inhibitory factor, is a cyclopeptide that plays an important role in inhibiting hormone secretion and neuronal excitability (Günther et al., 2018). Somatostatin receptor 4 (SSTR4) belongs to the SSTR family of G protein-coupled transmembrane receptors (GPCRs) comprised of five members (SSTR1–5), which trigger various transmembrane signaling pathways (Reisine and Bell, 1995; Csaba and Dournaud, 2001; Zou et al., 2019). *SSTR4* is expressed in areas involved in learning and memory processes (Günther et al., 2018). Gastambide et al. (2009) found that hippocampal *SSTR4* is functionally involved in a switch from hippocampus-based memory to dorsal striatum-based behavioral responses. Through a biological database, we found that *SSTR4* is highly expressed in brain tissue, and moderately expressed in the NAC, PFC, AMY and HPC. Psychostimulants are involved in the major brain regions including the ventral tegmental area (VTA), NAC, PFC, AMY, and HPC (Peña et al., 2014). Furthermore, a study by Moneta D indicated that SSTR4 enhanced (α-amino-3-hydroxy-5-methyl-4-isoxazoleioni-c axid, AMPA)-receptor-mediated excitatory signaling (Moneta et al., 2002) and that AMPA receptors were related to addiction (Godino et al., 2015). These results suggest that *SSTR4* may be related to reward and addiction. Temporal expression analyses showed that the expression level of *SSTR4* was relatively stable over time. However, our study showed hypomethylation of *SSTR4* in AD cases, which indicated a potential high expression of *SSTR4*. According to this, expression of *SSTR4* might be an upstream regulator of alcohol abuse, which can be inferred from previous findings, and suggests that alcohol abuse may ultimately affect *SSTR4* expression.

At present, there are few reports about the methylation of *SSTR4* related to AD. Dominika Berent interviewed 176 AD cases and 127 healthy controls to assess genotyping for the *SSTR4* rs2567608 polymorphism. The result revealed that AD cases and the controls did not differ significantly according to the *SSTR4* rs2567608 genotype and allele frequencies (Berent et al., 2017a). This study involved the relationship between the *SSTR4* genotype and AD, but did not examine the methylation of *SSTR4*. Another study interviewing the same participants revealed that the *SSTR4* promoter region was methylated in 21.6% of patients with AD and only 2.3% of controls (Berent et al., 2017b), suggesting a difference in methylation levels of *SSTR4* between AD cases and controls. This result is consistent with our present research in some respects.

The present study has several limitations. First, the sample size was relatively small, so further research will enlarge the sample size to verify the methylation levels of *SSTR4* by pyrosequencing. Second, we did not examine *SSTR4* expression levels in blood samples because they were unavailable for RNA extraction, and further research will analyze the correlation between DNA methylation and *SSTR4* expression in blood samples. Third, it still remains unclear whether epigenomic changes in peripheral cells could fully reflect the true DNA methylation status of brain. Nevertheless, tissue biopsies in every alcohol dependent subject are neither ethical nor practical, and previous studies have showed methylation of CpG positions occurring in PB might track part of the changes in central nervous system (Hillemacher et al., 2009). And last, AD in males were relatively easy to recruit, so subjects in this study were only males. But in view of the potential effect of gender on methylation, this may be a limitation of this study. In the further research, we may be recruit AD in females.

In summary, the promoter region of *SSTR4* differs between AD cases and controls. This study provides novel insights that heavy drinking likely results in alteration of epigenetic modification, which might in turn promote AD development. The hypothesis would integrate the understanding of methylation mechanism in the process of gene-environment interactions in alcohol-dependent patients. In addition, the *SSTR4* gene may represent a new biomarker for AD, which offers new ideas for the treatment of AD. Given these findings, additional effective therapeutic options may be developed in the future.

## Data Availability

The original contributions presented in the study are included in the article/Supplementary Material, further inquiries can be directed to the corresponding author.
